# Elevated levels of serum fatty acid synthase in patients with gastric carcinoma

**DOI:** 10.3892/ol.2014.1793

**Published:** 2014-01-14

**Authors:** TOMOAKI ITO, KOICHI SATO, HIROSHI MAEKAWA, MUTSUMI SAKURADA, HAJIME ORITA, KAZUNORI SHIMADA, HIROYUKI DAIDA, RYO WADA, MASAAKI ABE, OKIO HINO, YOSHIAKI KAJIYAMA

**Affiliations:** 1 Department of Surgery, Juntendo Shizuoka Hospital, Juntendo University School of Medicine, Shizuoka, Japan; 2 Department of Cardiovascular Medicine, Juntendo University School of Medicine, Tokyo, Japan; 3 Department of Pathology, Juntendo Shizuoka Hospital, Juntendo University School of Medicine, Shizuoka, Japan; 4 Department of Pathology and Oncology, Juntendo University School of Medicine, Tokyo, Japan; 5 Department of Esophageal and Gastroenterological Surgery, Juntendo University School of Medicine, Tokyo, Japan

**Keywords:** gastric cancer, fatty acid synthase

## Abstract

Gastric cancer is the second leading cause of cancer mortality in the world. It is important to develop biomarkers for detecting new cancers at an early stage and for treating them early during recurrence in order to guide optimal treatment. Fatty acid synthase (FAS) is highly expressed in numerous human cancers and thus could potentially serve as such a biomarker, but the potential utility of measuring FAS for detecting gastric cancer has not been previously investigated. The aim of the present study was to provide a preliminary assessment of serum FAS as a marker of gastric carcinoma. The study included 47 patients with gastric cancer and 150 healthy subjects. Blood samples were collected from each cancer patient prior to treatment. Serum FAS levels were measured by ELISA and compared across the two groups of patients. Significantly higher levels of serum FAS were found in the gastric cancer patients [95% confidence interval (CI), 30.37–52.46] compared with the healthy controls (95% CI, 1.331–2.131), with elevated levels even in patients with early-stage tumors. These results indicate that measuring serum FAS levels has strong potential to provide a biomarker for the detection of gastric cancer, with high sensitivity and specificity.

## Introduction

Gastric cancer is the second leading cause of cancer mortality in the world (1). In East Asia particularly, in countries such as China, Japan and Korea, more than one million new cases are diagnosed each year (2). As gastric cancer is associated with a short time to recurrence and a short survival period after recurrence, it is important to detect these cancers at an early stage and early on during recurrence to guide treatment of this disease (3). Effective biomarkers for detecting early-stage gastric cancer and recurrence could significantly aid its management.

A marker that is considered in the present study is fatty acid synthase (FAS), a metabolic enzyme that catalyzes the synthesis of long-chain fatty acids (4). FAS was first identified as oncogenic antigen 519 in patients with a poor prognosis for breast cancer (5). FAS is highly expressed in numerous human cancers, including cancer of the breast (6,7), prostate (8), colon (9), lung (10), bladder (11), ovary (12), stomach (13), esophagus (14), endometrium (15,16), pancreas (17,18) and kidney (19). Proliferating tumor cells use long-chain fatty acids for membrane assembly, lipid modifications of various proteins or as an efficient source of energy, all of which are necessary to sustain tumor growth and survival (20). The mechanism of FAS overexpression is unknown, however, it appears to be upregulated during the early stages of tumorigenesis (13,21,22), and levels of FAS overexpression generally correlate with tumor aggressiveness and poor prognosis (4,8,13,16,19,23–25).

Several studies have shown that serum FAS levels are increased in patients with breast (26,27), prostate (28), ovarian (28), colon (29) and pancreas (18) cancers compared with healthy controls. However, to date, there is no reported data regarding serum FAS level in patients with gastric cancer. The aim of the present study was to examine the expression of FAS in gastric cancer tissues by immunohistochemical staining, and to evaluate serum FAS as a potential marker of gastric cancer.

## Materials and methods

### Human subjects

The present study included 47 patients with gastric cancer (37 males and 10 females, aged 33–89 years old) who were diagnosed and had blood samples collected between January 2009 and September 2011 at Juntendo Shizuoka Hospital, Juntendo University School of Medicine (Shizuoka, Japan). All clinical diagnoses were confirmed by microscopic examination of the material obtained during surgery, endoscopic submucosal dissection or endoscopic biopsy. Fasting serum samples were collected from each cancer patient prior to treatment, and additionally, post-therapeutic samples were obtained from 11 of the patients with gastric cancer. In total, 35 of the 47 patients with gastric cancer underwent surgery, 10 underwent endoscopic submucosal dissection, and two underwent chemotherapy for unresectable tumors. The clinicopathological characteristics of these cases are summarized in Table I. Serum samples were also obtained from 150 healthy control individuals (113 males and 37 females, aged 33–83 years old) who underwent health screening at the Juntendo University School of Medicine. All patients and healthy volunteers provided signed informed consent prior to enrollment, and this study was approved by the Institutional Review Board of Juntendo Shizuoka Hospital, Juntendo University School of Medicine.

### Immunohistochemistry

Tissue sections, 3-μm thick, were prepared from archival formalin-fixed, paraffin-embedded specimens. Subsequent to deparaffinization, the tissue sections were heated in Target Retrieval Solution (DAKO, Glostrup, Denmark) for antigen retrieval and then treated with 3% hydrogen peroxide. Non-specific binding sites were blocked by incubation with 5% normal goat serum in phosphate-buffered saline (PBS) for 15 min at room temperature. The tissue sections were then incubated with anti-human FAS rabbit polyclonal antibody (Immuno-Biological Laboratories, Fujioka, Gunma, Japan) for 60 min at room temperature. The reaction was visualized using the EnVision+ System horseradish peroxidase-labeled polymer anti-rabbit antibody (DAKO, Glostrup, Denmark) and diaminobenzidine (Dojindo Laboratories, Tokyo, Japan) as the chromogen.

Analysis of FAS immunostaining was performed using a scoring system as described by Kusakabe *et al* (13). For the immunostained slides, the proportion of stained cancer cells was scored as 0 for <10%, 1 for 10–50% and 2 for >50%. The intensity was scored as 0 for no or very low intensity, 1 for moderate intensity, and 2 for high intensity, when compared to FAS-positive internal controls such as adipose tissue and peripheral nerve tissue. Using the sum of the two scores, positive FAS staining was defined as a score of ≥3.

### FAS ELISA

A total of 100 μl serum was analyzed using a commercially available ELISA kit, FAS-detect ELISA (FASgen, Immtech, Baltimore, MD, USA), according to the manufacturer’s recommendations. Sera were incubated in a 96-well capture plate on a plate shaker for 90 min at room temperature. The plate was then washed five times with wash buffer. FAS enzyme conjugate was added and the plate was incubated for 60 min, and the wash was repeated. Serum FAS levels were visualized by color change upon addition of tetramethylbenzidine substrate followed by addition of substrate stop solution. Absorbance was measured at 450 nm with a Benchmark Plus plate reader (Bio-Rad Laboratories, Hercules, CA, USA), and FAS concentrations were determined by interpolation from a standard curve.

### Statistical analysis

The data were analyzed with Graph Pad Prism 5.0 (GraphPad Software, San Diego, CA, USA). Measurement data were analyzed using the Mann-Whitney U test, Kruskal-Wallis test and Wilcoxon signed-rank test, whilst categorical data were analyzed using Fisher’s exact test. P<0.05 was considered to indicate a statistically significant difference. The receiver operator characteristic curve was used to determine sensitivity and specificity values.

## Results

### FAS expression in human gastric cancer

**Figure 1:**
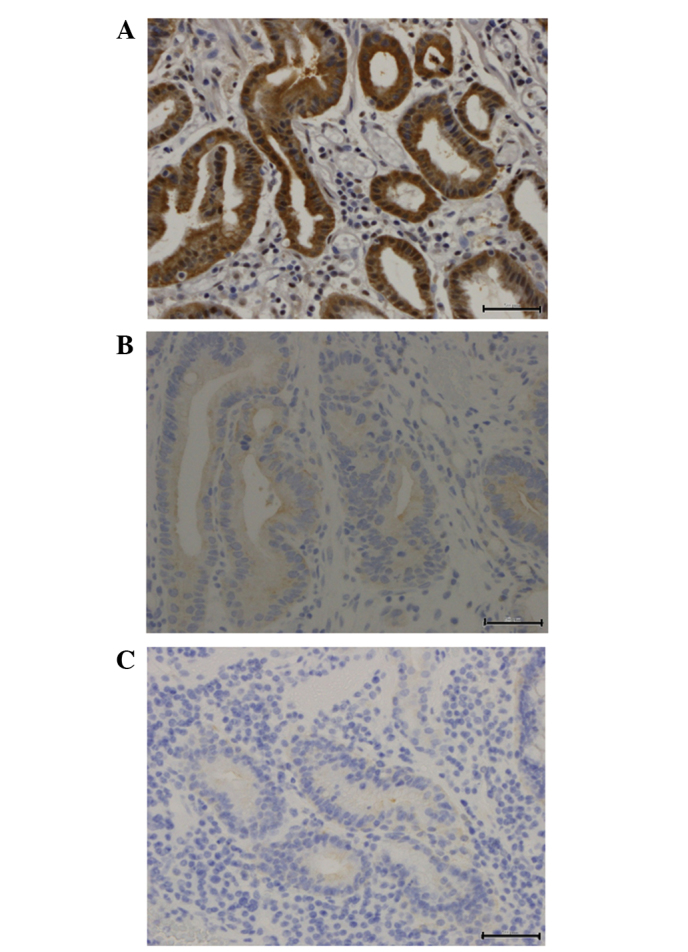
Immunohistochemical analysis. (A) Well-differentiated tubular adenocarcinoma cells strongly positive for FAS (intensity score, 2; proportion score, 2). (B) Cells moderately positive for FAS (intensity score, 1; proportion score, 1). (C) Cells negative for FAS. Scale bars, 50 μm. FAS, fatty acid synthase.

Immunohistochemically, FAS-positive staining was observed in the cytoplasm (Fig. 1) in 22 of 47 tumors, while 25 tumors were essentially negative for FAS staining. Higher levels of FAS expression were significantly associated with depth of carcinoma invasion (P=0.0423) and lymphatic invasion (P=0.0023). Correlations between the clinicopathological features and FAS expression in the primary tumors are summarized in Table I.

### Serum levels of FAS in patients with gastric cancer

**Figure 2:**
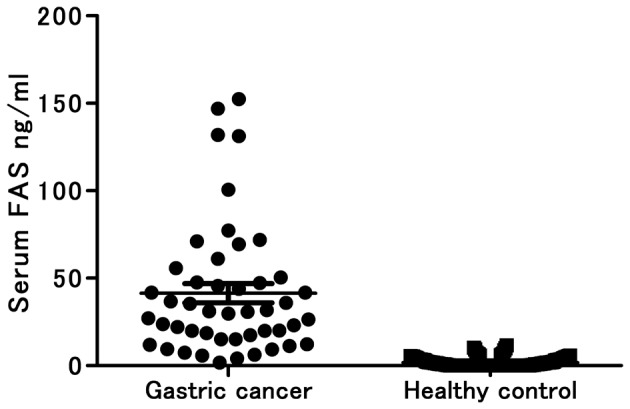
Serum FAS levels of gastric cancer patients were significantly higher than healthy controls (P<0.0001; Mann-Whitney U test). FAS, fatty acid synthase.

Overall, serum FAS levels were higher in the gastric cancer patients [95% confidence interval (CI), 30.37–52.46] than in the healthy controls (95% CI, 1.331–2.131) (P<0.0001; Fig. 2), and notably, more gastric cancer patients were found to have higher levels of serum FAS than predicted by the immunohistochemical staining of the tumor tissues. The best cutoff value that maximizes the sensitivity and specificity was 6.0 ng/ml. Using 6.0 ng/ml as the cutoff value, the sensitivity (93.62%) and specificity (93.33%) were the highest in the diagnosis of gastric cancer at optimal conditions, and the positive and negative predictive values were 81.48 and 97.90%, respectively (AUC, 0.9845; SE, 0.0084; 95% CI, 0.9681–1.001).

**Figure 3:**
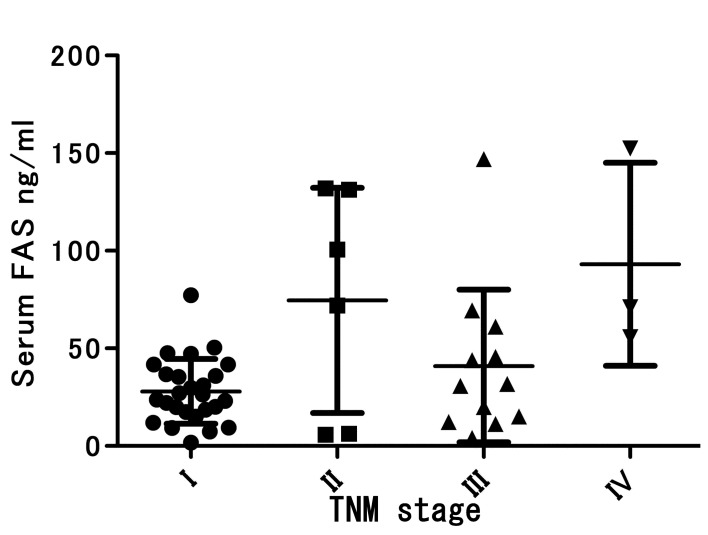
When the TNM staging was analyzed, there was no significance in serum FAS concentration (P=0.0603; Kruskal-Wallis test). TNM, tumor-node-metastasis; FAS, fatty acid synthase.

Unexpectedly, serum FAS levels did not correlate strongly with apparent tumor burden. For example, differences in serum FAS concentrations across tumor-node-metastasis (TNM) categories of tumors did not reach statistical significance (P=0.0603; Fig. 3). Notably, for each stage, the serum FAS levels were significantly higher in the cancer patients than in the healthy subjects (stage I, P<0.0001; stage II, P<0.0001; stage III, P<0.0001; and stage IV, P=0.0022). Given the low percentages of tumors with positive staining for FAS, it was not unexpected that there was no correlation between serum FAS concentration and immunohistochemical FAS staining (P=0.3763; Table II).

**Figure 4:**
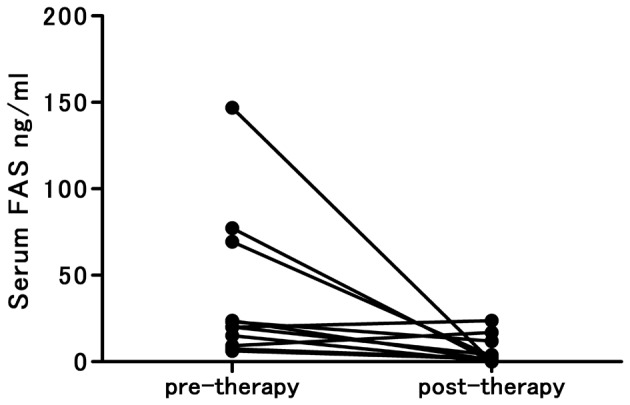
Comparison of pre-therapeutic and post-therapeutic serum FAS levels in gastric cancer patients (P=0.0098; Wilcoxon signed-rank test). FAS, fatty acid synthase.

The pre- and post- therapeutic levels of serum FAS were also compared for the patients with available specimens. In all such cases, the pre-therapeutic FAS levels were >6.0 ng/ml, and in 8 of 11 patients, the post-therapeutic serum FAS levels decreased to <6.0 ng/ml (P=0.0098; Fig. 4).

## Discussion

In the present study, FAS expression was examined in patients with gastric cancer by immunohistochemical staining of carcinoma tissue and through use of an ELISA to measure FAS levels in the serum of the patients. As aforementioned, positive staining for FAS was apparent in only 22 out of 47 cases (46.8%), but this high expression of FAS was associated with depth of invasion and lymphatic invasion, corroborating results of a previous study that reported that high FAS expression in gastric cancer tissues correlates with liver metastasis (13). However, the frequency of FAS-positive staining for gastric carcinoma tissue in the present study was higher than in the previous study (34.5%), and the present result was different from the previous result (13) that high FAS expression in gastric cancer tissues correlates with tumor differentiation. This indicates that the sample size in the present study was extremely small and the patients’ age was older (≥51 years old), with the exception of 3 patients. Kusakabe *et al* (13) demonstrated that the patients’ age correlated significantly with FAS status. FAS expression was frequently observed in carcinomas from the older age group (≥51 years old).

Although high FAS expression was most striking in the cancer tissues, the metaplastic, normal fundic and normal pyloric glands of the stomach also expressed detectable levels of FAS (data not shown). Hence, while not entirely specific for cancer, FAS does appear to be generally upregulated through various stages of gastric tumorigenesis (13,21,22).

Several previous studies have reported that serum FAS levels are higher in patients with breast, prostate, ovarian, pancreas and colorectal cancers (18,26–29), but serum FAS levels in patients with gastric cancer have not been previously analyzed. In the present study, the serum FAS levels of the gastric cancer patients were found to be significantly higher in comparison to the healthy controls, indicating that high concentrations of FAS in serum may result from enzyme secretion by cancer cells. Wang *et al* developed sandwich ELISA for the quantitative determination of FAS (28), and subsequently demonstrated that cultured breast cancer cells excrete immunoreactive FAS into the extracellular space and serum (26). In addition to finding increased levels of FAS in the majority of gastric cancer patients, the present study found that serum FAS levels decreased following therapy in 8 out of 11 gastric cancer patients with pre-and post- therapy measurements of FAS. Further evaluations of such patients will help to determine whether the extent of the decrease in serum FAS predicts recurrence. These findings are similar to those previously reported for pancreatic cancers, where FAS levels decreased following surgical resection of the tumor, indicating that the tumor was the primary source of circulating FAS in these patients (18).

In the present study, there was no significance in serum FAS concentration among the patients with various TNM-staged cancers. Rather, at each stage, serum FAS concentrations were significantly higher in the cancer patients than in the healthy subjects, indicating that serum FAS could aid in the early diagnosis of gastric cancer and that the determination of serum FAS concentration may possibly be used as a primary screening test for gastric cancer. Again, these findings are consistent with those reported for other types of cancers. For example, Notarnicola *et al* demonstrated that serum FAS levels in patients with colorectal cancer increased advancing clinical stage (29). On the other hand, Walter *et al* demonstrated that serum FAS was similarly elevated in pancreatic cancer patients, patients with intraductal papillary mucinous neoplasms and patients with chronic pancreatitis compared with healthy controls, indicating that FAS detection cannot be used for distinguishing pancreatic cancer patients from patients with other pancreatic diseases (18).

In conclusion, the present study is the first to compare the serum FAS levels of patients with gastric cancer and healthy subjects. Although a small sample size was used in this study, the data indicate that serum FAS has the potential to be useful as a biomarker for the detection of gastric cancer, with a sensitivity and specificity of 93.62 and 93.33%, respectively. This sensitivity result was higher than other classic tumor markers, including carcinoembryonic antigen, carbohydrate antigen 19-9 and carbohydrate antigen 72-4 (30,31). Further studies are required in order to compare the clinical significance of serum FAS with other classic tumor markers, and to determine whether serum FAS can be a useful biomarker for monitoring patients following treatment.

## Figures and Tables

**Table I:** Clinicopathological characteristics of patients with gastric cancer according to FAS staining.

		FAS status		
			
Parameter	No. of cases	Positive	Negative	P-value
Gender, n				
Male	37	18	19	
Female	10	4	6	0.7298
Age, years (mean ± SD)		72.3±13.2	72.2±11.0	0.5739
Maximal tumor size, mm (mean ± SD)		53.7±36.5	51.3±57.6	0.3285
Histology, n				
Well-moderate	32	12	20	
Poor	15	10	5	0.1155
Invasion depth, n				
T1	25	8	17	
T2–4	22	14	8	0.0423^a^
Lymph node metastasis, n^b^				
Positive	18	12	6	
Negative	27	10	17	0.0712
Lymphatic invasion, n^b^				
Positive	26	18	8	
Negative	19	4	15	0.0023^a^
Venous invasion, n^b^				
Positive	18	12	6	
Negative	27	10	17	0.0712
TNM stage, n				
1	26	9	17	
2–4	21	13	8	0.0823

a Statistically significant at P<0.05.

b Two cases undergoing chemotherapy without tumor resection were excluded.

SD, standard deviation; FAS, fatty acid synthase.

**Table II:** Correlation of FAS immunohistochemical expression with serum FAS.

	FAS, ng/ml (mean ± SD)	P-value
FAS IHC-positive	50.78±46.18	
FAS IHC-negative	33.17±26.34	0.3763

SD, standard deviation; IHC, immunohistochemistry; FAS, fatty acid synthase.
